# Associations between Taste Perception Profiles and Empirically Derived Dietary Patterns: An Exploratory Analysis among Older Adults with Metabolic Syndrome

**DOI:** 10.3390/nu14010142

**Published:** 2021-12-29

**Authors:** Julie E. Gervis, Rebeca Fernández-Carrión, Kenneth K. H. Chui, Jiantao Ma, Oscar Coltell, Jose V. Sorli, Eva M. Asensio, Carolina Ortega-Azorín, José A. Pérez-Fidalgo, Olga Portolés, Alice H. Lichtenstein, Dolores Corella

**Affiliations:** 1 Cardiovascular Nutrition Laboratory, Jean Mayer USDA Human Nutrition Research Center on Aging, Tufts University, Boston, MA 02111, USA; julie.gervis@tufts.edu (J.E.G.); alice.lichtenstein@tufts.edu (A.H.L.); 2 Department of Preventive Medicine and Public Health, University of Valencia, 46010 Valencia, Spain; rebeca.fernandez@uv.es (R.F.-C.); jose.sorli@uv.es (J.V.S.); eva.m.asensio@uv.es (E.M.A.); carolina.ortega@uv.es (C.O.-A.); j.alejandro.perez@uv.es (J.A.P.-F.); olga.portoles@uv.es (O.P.); 3 CIBER Fisiopatología de la Obesidad y Nutrición, Instituto de Salud Carlos III, 28029 Madrid, Spain; oscar.coltell@uji.es; 4 Department of Public Health and Community Medicine, Tufts University School of Medicine, Boston, MA 02111, USA; kenneth.chui@tufts.edu; 5 Department of Nutrition Data Science, Friedman School of Nutrition Science and Policy, Tufts University, Boston, MA 02111, USA; jiantao.ma@tufts.edu; 6 Department of Computer Languages and Systems, Universitat Jaume I, 12071 Castellón, Spain; 7 Department of Medical Oncology, Hospital Clínico Universitario Valencia, 46010 Valencia, Spain; 8 CIBERONC, Instituto de Salud Carlos III, 28029 Madrid, Spain

**Keywords:** taste, taste perception, dietary patterns, sweet, salt, sour, bitter, umami, data-driven, individual differences, personalized nutrition

## Abstract

Taste perception is a primary driver of food choices; however, little is known about how perception of all five tastes (sweet, salt, sour, bitter, umami) collectively inform dietary patterns. Our aim was to examine the associations between a multivariable measure of taste perception—taste perception profiles—and empirically derived dietary patterns. The cohort included 367 community-dwelling adults (55–75 years; 55% female; BMI = 32.2 ± 3.6 kg/m^2^) with metabolic syndrome from PREDIMED-Plus, Valencia. Six taste perception profiles were previously derived via data-driven clustering (Low All, High Bitter, High Umami, Low Bitter and Umami, High All But Bitter, High All But Umami); three dietary patterns were derived via principal component analysis (% variance explained = 20.2). Cross-sectional associations between profiles and tertials of dietary pattern adherence were examined by multinomial logistic regression. Overall, there were several significant differences in dietary pattern adherence between profiles: the vegetables, fruits, and whole grains pattern was significantly more common for the High All But Umami profile (OR range for high vs. low adherence relative to other profiles (1.45–1.99; 95% CI minimum lower, maximum upper bounds: 1.05, 2.74), the non-extra virgin olive oils, sweets, and refined grains pattern tended to be less common for Low All or High Bitter profiles (OR range: 0.54–0.82), while the alcohol, salty foods, and animal fats pattern tended to be less common for Low Bitter and Umami and more common for High All But Bitter profiles (OR range: 0.55–0.75 and 1.11–1.81, respectively). In conclusion, among older adults with metabolic syndrome, taste perception profiles were differentially associated with dietary patterns, suggesting the benefit of integrating taste perception into personalized nutrition guidance.

## 1. Introduction

Over the past few decades, efforts to promote healthy dietary patterns have had limited impact [1]. Globally, suboptimal diet quality has become a leading risk factor for cardiometabolic diseases [2] across age, sex, and socioeconomic groups [3]. From a public health perspective, this has created a need for more effective dietary modification efforts which can better promote health and reduce the burden of diet-related chronic diseases.

Traditionally, dietary modification efforts have primarily employed “one size fits all” approaches through the dissemination of population-wide dietary guidelines [4]. Alternatively, there has been mounting interest in using more personalized nutrition approaches, which leverage information on individual characteristics to develop targeted advice which may be more effective [5,6]. Support for this approach has been accumulating [7], specifically for guidance tailored to individuals’ responses to food [8], dietary habits [9], or genetic traits [10]. Less studied, however, are the individual-level drivers of food choices which may better promote long-term adherence to personalized diet advice [11].

Among the myriad factors driving food choices is taste perception—an individual’s ability to perceive the five basic tastes: sweet, salt, sour, bitter, and umami. Evolutionarily, perception of all five tastes was vital for mediating nutrient intakes and protecting against noxious substances [12,13]. In the modern food environment, individual differences in taste perception have been related to preferences for and intakes of various food groups. For example, differences in bitter, sweet, or salt perception have been independently associated with intakes of cruciferous vegetables [14,15,16,17], desserts and sugar-sweetened beverages (SSBs) [18,19], red meat and olive oil [20], and alcoholic beverages [21]. However, the dearth of evidence on the relations of sour and umami perceptions with food choices, and the paucity of studies evaluating the simultaneous associations between perception of multiple tastes and dietary intake, limit our ability to leverage these individual traits when developing personalized nutrition guidance aimed at reducing chronic disease risk.

Consistent with the shift towards studying dietary patterns rather than single foods or nutrients to capture the overall effects of diet on health outcomes [22,23], one approach to better capture the relations between taste perception and diet may be to study “taste perception profiles”, a multivariable measure of perception for all five tastes, rather than considering each taste separately. Previously, we demonstrated that a data-driven clustering approach could be used to derive valid and stable taste perception profiles from sweet, salt, sour, bitter, and umami taste perception scores. Using this approach, we identified six distinct taste perception profiles among a cohort of community-dwelling older adults with metabolic syndrome [24]. A critical next step for this work is to evaluate dietary behaviors among individuals with different taste perception profiles [25] to determine whether individuals with similar taste perception profiles make similar food choices. Therefore, our present aim was to explore the associations between taste perception profiles and habitual dietary patterns. To accomplish this, we derived empirical dietary patterns from habitual food group intakes and examined the associations between taste perception profiles and adherence to these dietary patterns, using cross-sectional data from community-dwelling older adults with metabolic syndrome.

## 2. Materials and Methods

### 2.1. Study Design and Participants

The present study was a site-specific project in the PREDIMED (PREvención con DIeta Mediterránea)-Plus Valencia study. PREDIMED-Plus is an ongoing, multicenter, randomized controlled trial testing the effect of an energy-restricted Mediterranean diet coupled with a physical activity intervention for the primary prevention of cardiovascular disease, compared to a control group [26]. PREDIMED-Plus Valencia was one of the recruiting centers and the only site conducting taste perception tests. Participants were recruited to be high risk community-dwelling older women (60–75 years) and men (55–75 years) residing in Spain, with elevated BMI (>27 and <40 kg/m^2^) and metabolic syndrome [26,27]. In total, 381 participants completed the taste perception tests at baseline [28], of whom, 2 were excluded for implausible total energy intake determined based on prior studies (>4000 kcal/day for women and >4500 kcal/day for men) [29] and 12 for incomplete data for some taste perception tests, resulting in a sample size of 367 for this cross-sectional analysis (see flow chart in Figure S1).

All participants provided written informed consent in accordance with ethical standards of the Declaration of Helsinki. PREDIMED-Plus study protocols were approved by the Human Research Ethics Committee of Valencia University, and the current analysis by the Tufts University Institutional Review Board.

### 2.2. Anthropometric and Biochemical Parameters

Anthropometric and biochemical parameters were measured by trained study staff in accordance with PREDIMED-Plus operations protocols [26]. Weight and height were measured using calibrated scales and a wall-mounted stadiometer, respectively. Waist circumference was measured with anthropometric tape after normal exhalation. Blood pressure was measured using a validated semiautomatic oscillometer (Omron HEM-705CP), in triplicate, in a seated position after 5 min of rest. Fasting blood glucose and plasma triglyceride and total, HDL, and LDL cholesterol (HDL-c and LDL-c, respectively) concentrations were measured using samples collected after a 12 h overnight fast, as previously described [30].

### 2.3. Taste Perception Assessment

Taste perception was assessed by trained technicians in a standardized environment as previously described, in detail [28]. Briefly, one representative tastant was used for each of sweet, salt, sour, and umami (sucrose, NaCl, citric acid, and monopotassium L-glutamate (MPG), respectively); two representative tastants were used for bitter (phenylthiocarbamide (PTC) and 6-n-propylthiouracil (PROP)). Tastants were provided in a standardized order (sucrose, NaCl, citric acid, MPG, PTC, PROP) to minimize context effects. All tastant solutions were prepared in distilled water. Sweet, salt, sour, and umami solutions were provided using a wooden cotton bud applicator and bitter solutions were provided on solution-soaked filter papers. Each tastant was applied to the tongue, and the participants were instructed to mix them with saliva for 30 s then spit them out. After which, participants rated the intensity of each solution using a categorical scale which had been previously validated in this population [28] and was chosen instead of a general Labeled Magnitude Scale (gLMS) because of its simplicity which may make it easier to understand. The scale ranged from 0 to 5: 0 = “no taste”, 1 = “weak”, 2 = “moderate”, 3 = “strong”, 4 = “very strong”, 5 = “extremely strong.” On this basis, higher taste perception scores indicated greater perception intensity of the tastant solutions. Of note, although both PTC and PROP were used to assess bitter taste perception, only PTC was used to represent bitter taste in the taste perception profiles so that each taste could be represented by only one tastant and PTC was provided before PROP in the taste perception tests. Additionally, although five solutions of increasing concentrations were used per tastant [28], only the most concentrated solutions (400 mM sucrose, 200 mM NaCl, 34 mM citric acid, 200 mM MPG, and 5.6 mM PTC, respectively) were used to derive the taste perception profiles because they elicited the highest inter-individual variability in taste perception scores.

### 2.4. Taste Perception Profiles

**Figure 1:**
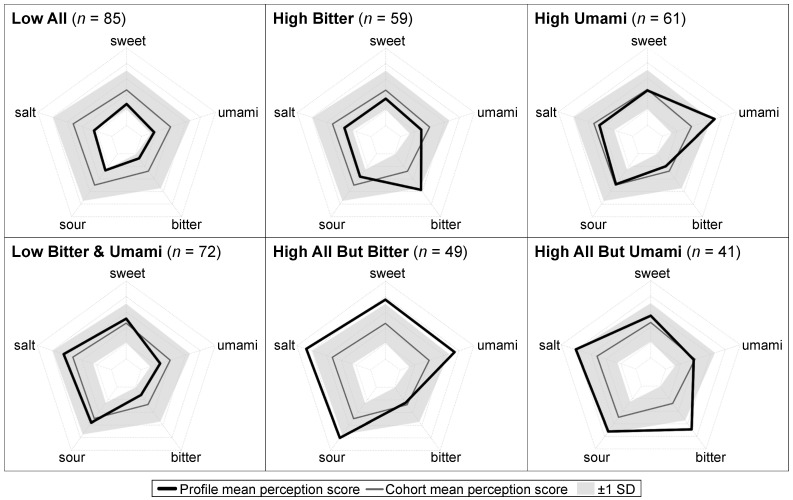
Six taste perception profiles derived via a data-driven clustering approach in the PREDIMED-Plus Valencia cohort; *N* = 367 (adapted with permission [24]). Mean perception of each taste for each profile is depicted in solid black lines; mean ± 1 SD perception of each taste for the overall cohort is represented by dark gray lines and shaded areas, respectively. Taste perception scores ranged from 0–5; 0 is the innermost pentagon and 5 is the outer most pentagon.

Taste perception profiles were previously derived from sweet, salt, sour, bitter, and umami perception scores using a data-driven clustering approach, as described in detail [24]. Briefly, cluster analysis was used to identify sub-groups of individuals with a common taste perception profile. The selection of the cluster algorithm, number of clusters, and specific set of clusters (herein referred to as taste perception profiles) was informed by quantitative criteria to limit subjectivity. Based on the criteria, a k-means clustering algorithm was selected and six taste perception profiles were derived. Radar plots were used to visualize the characteristics of each profile relative to the overall cohort. The six taste perception profiles were: Low All (*n* = 85, 23%), High Bitter (*n* = 59, 16%), High Umami (*n* = 61, 17%), Low Bitter and Umami (*n* = 72, 20%), High All But Bitter (*n* = 49, 13%) and High All But Umami (*n* = 41, 11%) (Figure 1). For example, the Low All profile was characterized by mean perception scores for all five tastes roughly 1 SD below the cohort means, while the High All But Umami profile was characterized by mean perception scores for four tastes roughly 1 SD above the cohort means with a mean perception score for umami close to the cohort mean. As previously reported, all taste perception profiles had high internal validity and stability and were well-fitted to the data [24]. 

### 2.5. Dietary Assessment

Participants’ dietary intake during the prior year was assessed at baseline using a validated, culturally specific, 143-item semi-quantitative food frequency questionnaire (FFQ), in Spanish [31]. The FFQ was administered via face-to-face interviews. Nine food frequency categories were offered ranging from never or <1 serving per month to ≥6 servings per day. These data were then converted into number of servings per week for all subsequent analyses. FFQ items consumed by fewer than 5% of participants were not considered part of the habitual diet for the cohort and were omitted from analyses (14 items).

### 2.6. Empirically Derived Dietary Patterns

Empirically derived dietary patterns were identified using principal component analysis (PCA). PCA was selected because it is the most widely used approach for deriving *a posteriori* dietary patterns in nutritional epidemiology. Briefly, PCA is a dimension reduction technique that seeks to identify the fewest number of robust latent factors (i.e., dietary patterns) which capture the most variability in the original data (i.e., food group intakes). First, FFQ data were collapsed into 40 food groups, defined based on nutrient composition, culinary usage, and with consideration of food items’ taste (e.g., cruciferous (Brassica) vegetables were separated from green leafy and red/orange vegetables, and citrus fruits were separated from non-citrus fruits) (Table S1). Since a majority of food groups were non-normally distributed, a square root transformation was applied to the data prior to all statistical analyses. Prior to performing PCA, food groups were energy adjusted using the residual method, by regressing each food group onto total energy intake [32]. Model residuals were extracted then entered into PCA. Eigenvalues (>2), the break in the Scree plot, and the interpretability of identified patterns were used to determine the number of patterns to retain. A Varimax rotation was then applied to the identified patterns to increase separation and improve interpretability [33]. Derived patterns (herein referred to as empirically derived dietary patterns) were labeled according to which food groups had high (>|0.20|) rotated factor loadings, consistent with prior work [34,35].

For each participant, an adherence score was computed for each empirically derived dietary pattern by summing their reported food group intakes weighted by the food group’s factor loading for each respective dietary pattern, as follows:

(1) $$Adherence\;score\;for\;dietary\;pattern\;i=\sum [Intake_{j}\times Factor\;Loading_{ji}]$$

where *Intake_j_* is the reported intake (servings/week) of the *j*th food group and *Factor Loading_ji_* is the PCA-derived factor loading for the *j*th food group on the *i*th empirically derived dietary pattern [22,33]. On this basis, higher adherence scores reflected greater alignment to the empirically derived dietary patterns (i.e., food groups with positive factor loadings were consumed more often and food groups with negative factor loadings were consumed less often) and vice versa. Based on these calculations, participants were classified into tertials of adherence for each dietary pattern: scores in tertial 1 were considered “low adherence”, scores in tertial 2 were considered “moderate adherence”, and scores in tertial 3 were considered “high adherence.” For the primary analysis, only the extreme comparisons (high vs. low adherence) were conducted. This approach was taken to provide greater discriminatory power and to allow for comparisons to be conducted among those with the greatest differences in dietary patterns.

### 2.7. Covariates

Covariates were selected *a priori* based on previously established relations with the exposures and outcomes of interest. Demographic and lifestyle measures, such as age, sex, smoking status and history, and medication use, were assessed via participant questionnaires, described previously [28]. Physical activity in metabolic equivalents (METs) per week was estimated using a validated questionnaire [36]. Type 2 diabetes status was defined as having a clinical diagnosis of type 2 diabetes, use of glucose medications (insulin or Metformin), or fasting blood glucose ≥7.0 mmol/L [30]. BMI was calculated from weight and height in kg/m^2^. Regarding energy intake, given the cross-sectional study design, an *a priori* decision was made to model it as downstream of dietary patterns rather than as a confounder of taste-diet relations. On this basis, energy intake was not treated as a covariate in primary analyses, though it was explored as a covariate in sensitivity analyses to rule out the potential for confounding.

### 2.8. Statistical Analysis

Descriptive statistics—expressed as means ± SDs or *n* (%) unless otherwise specified—were used to examine central tendency and dispersion of demographic, clinical, and lifestyle characteristics overall and according to taste perception profile and level of adherence to each empirically derived dietary pattern. Variable distributions were examined for normality both visually, using histograms, and statistically, using Shapiro–Wilk tests. Violations were addressed via appropriate transformations. ANOVA tests were used to evaluate differences in means of continuous variables; chi-square tests were used to evaluate differences in proportions of categorical variables. When ANOVA *p*-values < 0.05, Student’s *t-*tests were used for *post hoc* pairwise comparisons of continuous variables.

Primary statistical analyses included generalized linear models with taste perception profiles treated as a 6-level categorical predictor. All regression models were adjusted for covariates, such as age, sex, physical activity (METs/wk), smoking status (current/non-smoker), medication use (antihypertensive and cholesterol lowering as two separate covariates), type 2 diabetes, and BMI. A BMI^2^ term was also entered into the model based on observed non-linear trends in BMI in preliminary regression models.

To examine differences in dietary pattern adherence across individuals with different taste perception profiles, multinomial logistic regression models were employed using low adherence and each taste perception profile as reference groups. On this basis, models were used to estimate the adjusted odds ratios (ORs) and corresponding 95% CIs of having high, relative to low, adherence to each empirically derived dietary pattern for individuals with each taste perception profile, relative to other taste perception profiles. Likelihood ratio tests were then used to examine the overall or “joint” significance of relations between taste perception profiles and adherence to each empirically derived dietary pattern by determining whether fully adjusted regression models, including all taste perception profiles and covariates, outperformed regression models including covariates alone. 

To rule out the potential for confounding by energy intake, *post hoc* sensitivity analyses were conducted by entering an energy intake term (kcal/day) as a covariate into each of the multinomial logistic regression models. Regression estimates and model fit statistics were subsequently evaluated and compared.

All statistical analyses were conducted in R (*v*3.5.1). Two-sided *p*-values < 0.05 were considered statistically significant. Given the exploratory nature of this analysis and small sample size, no corrections were made for multiple comparisons as this may increase the risk of obtaining false negative results.

## 3. Results

### 3.1. Participant Characteristics by Taste Perception Profile

The participant characteristics were consistent with the recruitment criteria (22). Their mean age was 65 ± 5 years (mean ± SD), their mean BMI was 32.3 ± 3.6 kg/m^2^ and they had several metabolic syndrome risk factors (Table 1).

When stratifying by taste perception profile, participants with a Low All profile were most likely to be male, current or former smokers, and to have type 2 diabetes (all *p* < 0.05) (Table 1). Those with a High All But Bitter or High All But Umami profile were most likely to be female and to have never smoked. Those with a High All But Umami profile were also least likely to have type 2 diabetes (*p* = 0.011) and they had the highest mean total and LDL-c concentrations, while those with a High All But Bitter or Low Bitter and Umami profile had the lowest mean total and LDL-c concentrations (*p* = 0.011 and 0.037, respectively). Blood pressure and fasting blood glucose, triglyceride, or HDL-c concentrations were similar across participants with different taste perception profiles.

### 3.2. Empirically Derived Dietary Patterns

**Figure 2:**
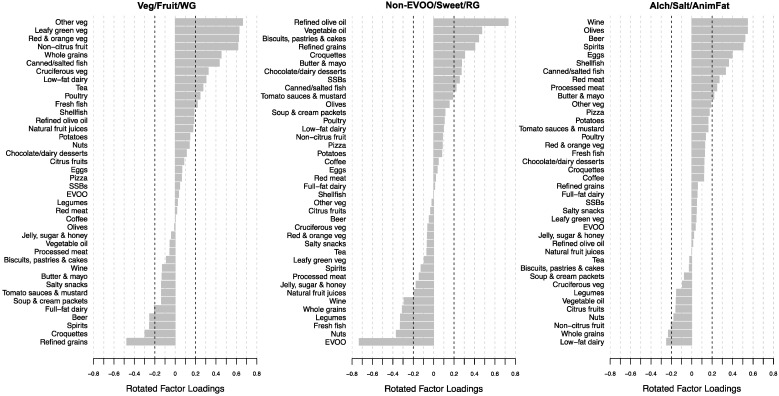
Rotated food group factor loadings from principal component analysis for three empirically derived dietary patterns. EVOO, extra virgin olive oil; SSBs, sugar-sweetened beverages.

Three empirically derived dietary patterns were identified with eigenvalues >2, altogether, accounting for 20.2% of total variance in food group intakes (Figure 2, Figure S2 and Table S2). The first dietary pattern, termed “Vegetables, Fruits, and Whole Grains” (Veg/Fruit/WG), accounted for 8.6% of total variance. It was characterized by high positive factor loadings for other vegetables, leafy green vegetables, red and orange vegetables, non-citrus fruits, whole grains, canned and salted fish, cruciferous vegetables, low-fat dairy, tea, poultry and fresh fish, and high negative factor loadings for refined grains, croquettes, spirits, and beer. The second dietary pattern, termed “Non-Extra Virgin Olive Oils (EVOOs), Sweets, and Refined Grains” (Non-EVOO/Sweet/RG), accounted for 6.3% of total variance. It was characterized by high positive factor loadings for refined olive oil, vegetable oil, biscuits, pastries and cakes, refined grains, croquettes, butter and mayonnaise, chocolate and dairy desserts, SSBs, and canned and salted fish, and high negative factor loadings for EVOO, nuts, fresh fish, legumes, whole grains, and wine. The third dietary pattern, termed “Alcohol, Salty Foods, and Animal Fats” (Alch/Salt/AnimFat), accounted for 5.3% of total variance. It was characterized by high positive factor loadings for wine, olives, beer, spirits, eggs, shellfish, canned and salted fish, red meat, processed meat, and butter and mayonnaise, and high negative factor loadings for low-fat dairy and whole grains. Only two food groups had high positive factor loadings on multiple dietary patterns (canned and salted fish, and butter and mayonnaise), indicating strong separation between patterns.

### 3.3. Participant Characteristics by Level of Adherence to Empirically Derived Dietary Patterns

Several participant characteristics differed significantly across levels of adherence to the empirically derived dietary patterns (Table 2). Participants with high adherence to the Veg/Fruit/WG dietary pattern were most likely to be female and older (*p* = 0.01 and 0.018, respectively), have never smoked (*p* = 0.012), and yet, have higher LDL-c concentrations (*p* = 0.038). Participants with high adherence to the Non-EVOO/Sweet/RG dietary pattern were least physically active (*p* = 0.003) and most likely to have type 2 diabetes and report the use of glucose control medications (*p* = 0.007 and 0.009, respectively). In contrast, participants with high adherence to the Alch/Salt/AnimFat dietary pattern were most likely to be male and younger (*p* ≤ 0.001 and 0.004, respectively), be a current or former smoker (*p* < 0.001) and have the highest diastolic blood pressure and total cholesterol concentrations (*p* = 0.013 and 0.047, respectively).

### 3.4. Association between Taste Perception Profiles and Empirically Derived Dietary Patterns

**Figure 3:**
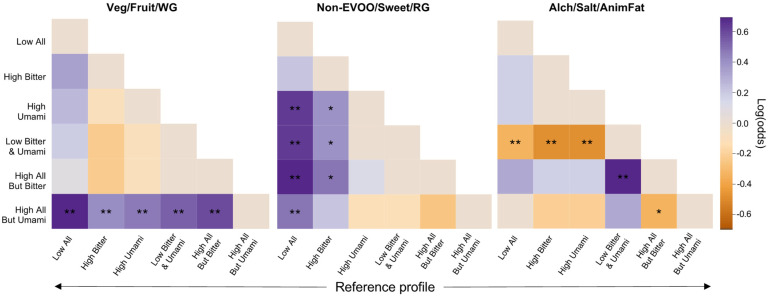
Heat map showing the odds (in log(odds)) of having high, relative to low, adherence to each empirically derived dietary pattern among participants with each taste perception profile (*N* = 367 participants included in each heat map). Regression models were adjusted for age, sex, physical activity, smoking status, medication use, type 2 diabetes, and BMI. The profiles on the vertical axis are the test profiles and those on the horizontal axis are the reference profiles; together they lay the grid for all unique pairwise comparisons. Colors indicate the magnitude of association; darker purple indicates higher odds of having high adherence and darker orange indicates lower odds of having high adherence relative to the reference profile. For example, in the left panel, the dark purple square at the lower left indicates that relative to individuals with a Low All profile (reference), those with a High All But Umami profile had significantly higher odds of having high, relative to low, adherence to the Veg/Fruit/WG dietary pattern. * *p* < 0.05, ** *p* < 0.01.

The regression models which included all six taste perception profiles (in addition to the other covariates), did not significantly outperform models that included the covariates alone (likelihood ratio test *p*-values = 0.631, 0.372, 0.350 for the Veg/Fruit/WG, Non-EVOO/Sweet/RG, and Alch/Salt/AnimFat dietary patterns, respectively) (see full regression results in Tables S3–S5). Although this suggested a non-significant joint association between all six taste perception profiles and adherence to the empirically derived dietary patterns, this may result from the relatively small sample or profile sizes, which could limit discriminatory power. Nonetheless, in the fully adjusted models, pairwise comparisons between individuals with different taste perception profiles identified several significant differences in adherence to all three empirically derived dietary patterns, indicating potentially important trends to explore (Figure 3).

For the Veg/Fruit/WG dietary pattern (Table S3), high (relative to low) adherence was significantly more common among participants with a High All But Umami profile relative to those with any other profile (range of ORs: 1.45–1.99; 95% CI minimum lower bound: 1.05, maximum upper bound: 2.74; all *p* < 0.05), with greatest differences observed relative to those with a Low All profile (OR (95% CI) for High All But Umami vs. Low All: 1.99 (1.55, 2.74); *p* < 0.001). For the Non-EVOO/Sweet/RG dietary pattern (Table S4), participants with a Low All profile were significantly less likely to have high adherence than those with all other profiles except High Bitter, who likewise tended to have low adherence (range of ORs: 0.55–0.66; 95% CI minimum lower bound: 0.40, maximum upper bound: 0.88; all *p* < 0.01). Finally, for the Alch/Salt/AnimFat dietary pattern (Table S5), participants with a High All But Bitter profile were most likely to have high adherence, though they were only significantly more likely than those with a Low Bitter and Umami or High All But Umami profile (OR (95% CI) = 1.81 (1.33, 2.47), *p* < 0.001 and 1.37 (1.01, 1.85), *p* = 0.045, respectively). In contrast, participants with a Low Bitter and Umami profile were least likely to have high adherence, and they were significantly less likely than those with all other profiles except High All But Umami, who likewise tended to have low adherence (range of ORs: 0.55–0.71; 95% CI minimum lower bound: 0.40, maximum upper bound: 0.92; all *p* < 0.05). These data indicate that a healthier dietary pattern, the Veg/Fruit/WG pattern, was most common among participants with a High All But Umami profile, whereas less healthy dietary patterns, such as the Non-EVOO/Sweet/RG or Alch/Salt/AnimFat patterns, tended to be more common among those with a High All But Bitter profile and less common among those with a Low All, High Bitter, or Low Bitter and Umami profile.

In *post hoc* sensitivity analyses, no evidence of confounding by energy intake was detected (mean ± SD % change in ORs: 4.55 ± 3.36), nor did the addition of an energy intake term substantially improve the predictability of regression models (data not shown). On this basis, no revisions were made to the original causal diagram, and hence, no additional adjustment was made for energy intake in the final statistical models.

## 4. Discussion

Using taste perception profiles previously derived in the PREDIMED-Plus Valencia cohort of community-dwelling older adults with metabolic syndrome [24], this cross-sectional exploratory analysis found that adherence to three empirically derived dietary patterns—Veg/Fruit/WG, Non-EVOO/Sweet/RG, Alch/Salt/AnimFat—tended to differ among participants with different taste perception profiles. After adjusting for confounders, participants with high perception of all tastes except umami were most likely to report following a healthier, prudent-style dietary pattern [4,37,38] characterized by frequent intake of a variety of vegetables, fruits, whole grains, and low-fat dairy, and less frequent intake of refined grains and products made thereof. In contrast, those with high perception of all tastes except bitter were more likely to report following a less healthy, Western-style dietary pattern [4,37,38] characterized by frequent intake of wine, beer and spirits, salty foods, and red and processed meats, and less frequent intake of whole grains and low-fat dairy. Participants with lower taste perceptions were less likely to follow unhealthy dietary patterns; instead, those with low overall or only high bitter perception tended to report frequent intakes of legumes, fresh fish, and nuts, while those with low bitter and umami but average sweet, salt, and sour perception tended to report more frequent intakes of whole grains and low-fat dairy.

Although statistically significant joint associations between the taste perception profiles and empirically derived dietary patterns were not detected, the observed between-profile differences in dietary pattern adherence may be of clinical relevance. In this cohort, individuals with similar taste perception profiles tended to follow more similar dietary patterns relative to those with different taste perception profiles. From a clinical perspective, these findings suggest that taste perception profiles may serve as an additional tool to promote adherence to personalized nutrition recommendations by identifying target areas for improvement. Based on the present findings, this could involve emphasizing recommendations to choose poultry or fish over red and processed meats and limit high sodium foods for individuals with high perception of all tastes except bitter who tended to follow the Alch/Salt/AnimFat pattern; and emphasizing recommendations to choose whole over refined grains and consume a variety of vegetables and fruits for those with low perception of all five tastes who tended not to follow the Veg/Fruit/WG pattern [4]. 

Previous studies have reported several associations between taste perception and food intake [14,15,16,17,18,19,20,21,39]. In adults, higher bitter perception has been associated with lower intake of vegetables [14,15,16], alcoholic beverages [21], and sweets [17]. A recent study also found that that higher umami perception was associated with higher intake of vegetables [39]. However, several findings in the present study were not consistent with those previously reported on taste perception and diet [14,15,16,17,18,19,20,21,39]. In this study, participants with high bitter perception and moderate umami perception—those with a High All But Umami profile—were more likely to follow a vegetable-rich dietary pattern than those with lower bitter or higher umami perception. They were also more likely to follow a dietary pattern with frequent wine, beer, or spirits than those with a Low Bitter and Umami profile who had lower bitter perception. Although, as expected, participants with high bitter perception (High Bitter profile) were less likely to follow a dietary pattern rich in sweets and desserts than most others, in this cohort, participants with lower bitter perception (Low All profile) unexpectedly followed similar dietary habits.

A potential explanation for these new findings may be that the multivariable measure of perception for all five tastes used in this study allowed for the capture of different aspects of taste-diet relations than prior studies which evaluated each taste separately. Myriad taste compounds are present in foods and beverages. Thus, considering all five tastes simultaneously when relating taste perception to diet may provide a more comprehensive representation of taste-diet relations—similarly to how dietary patterns better represent overall diet quality than single food groups or nutrients [22]. Multivariable approaches can also account for within-individual variability in taste perception, whereas these underlying differences may confound relations emanating from single taste studies or those using total taste scores (sum of multiple taste perceptions) [24].

Of note, the specific taste perception profiles and dietary patterns identified in the PREDIMED-Plus cohort of older, overweight adults with metabolic syndrome may differ from those found in cohorts of younger, healthier adults with different lifestyle characteristics [28,40]. Taste perception profiles and associations with dietary patterns may also be sex specific. In this cohort, taste perception profiles and levels of dietary pattern adherence differed significantly by sex, similar to prior reports [37,41]. *Post hoc* exploratory analyses further suggested several interactions by sex in regression models predicting dietary pattern adherence; however, there was insufficient statistical power to conduct stratified analyses by sex in this study. Additionally, in contrast to many Westernized countries, this cohort does not consume large quantities of processed hyperpalatable foods [42], which may have influenced their taste perception profiles and food choices. To fully understand the impact of taste perception profiles on diet quality will require the replication of this analysis in multiple, diverse cohorts and in various food environments.

Limitations of this study include the cross-sectional design which prevents causal inferences and the self-reported nature of the dietary data which may be subject to measurement errors. However, the dietary data were collected using a validated and culturally specific FFQ which was specifically designed to estimate habitual intake in this cohort [31]. While the percent variance explained by the dietary patterns may not appear high, it is similar to prior studies [33,43] and expected given that adjusting food groups for energy prior to PCA may attenuate the variance explained [44]. While the lack of energy adjustment in statistical models may increase the risk of measurement error [32], the intent was to focus on *ad libitum* intakes as opposed to dietary composition at isocaloric levels. Still, to reduce the risk of measurement error, BMI was controlled for in all models as a more objective indicator of misreporting than energy derived from an FFQ [45]. All food groups were also energy-adjusted prior to PCA, and in *post hoc* sensitivity analyses, energy did not appear to confound observed taste-diet relations. The lack of adjustment for multiple comparisons may have also increased the potential for spurious associations. However, this study was exploratory in nature and intended to generate hypotheses to be tested in larger, more representative cohorts. 

In the PREDIMED-Plus study, perception intensity rather than sensitivity (e.g., detection or recognition thresholds), was assessed given prior evidence that it may better capture relations with dietary intake [46,47]. To measure perception intensity, a six-point categorical scale was used rather than a gLMS with cross-modality standards because it was judged as more suitable for older adults given its simplicity which makes it easier to understand [48]. The scale was also validated in this population (dose-response, internal consistency, and association with genetic markers) [28]; and when used in a GWAS of taste perception, both PTC and PROP scores successfully identified top-ranked SNPs in the TAS2R38 bitter taste receptor gene which have been previously identified using gLMS [49,50]. This suggested that the category scale was valid and comparable to the gLMS for detecting individual differences in taste perceptions. PTC and PROP were the bitter tastants used in the parent study since they are the most frequently used bitter tastants in the literature [51,52]. Had other tastants for bitter been used, such as quinine or caffeine, the results may have been different [53]. Only PTC was used to derive the taste perception profiles because one tastant was chosen per taste and PTC was the first bitter stimuli to which participants were exposed. However, it is unlikely this selection impacted the results given the strong correlation between PTC and PROP taste perceptions [54]. Also, although all 5 basic tastes were included in the taste perception profiles, one potential limitation is the lack of data for what has been suggested as a sixth taste for oral fat sensitivity [55,56] which was not feasible to assess in the parent study. 

As previously reported, all six taste perception profiles were statistically valid when assessed by rigorous quantitative criteria; and when bootstrapping was applied to generate 100 permutations of the data, identical profiles were detected 78–91% of the time (Jaccard similarity index range: 0.78–0.91) [24]. However, we cannot rule out the possibility that the profiles identified in the PREDIMED-Plus Valencia cohort do not represent those in other cohorts with other dietary patterns. That assessment awaits future work. As all participants in the PREDIMED-Plus cohort had metabolic syndrome, they were all at elevated cardiometabolic risk. This diminished the variability among participants, hence, the ability to detect associations between dietary patterns and cardiometabolic risk factors. Nevertheless, the cohort represents a clinically relevant group and the use of empirical, rather than *a priori*, dietary patterns allowed for a focus on actual food intake.

Notwithstanding these limitations, this study is the first to examine how individual differences in perception of all five tastes collectively, rather than separately, relate to empirically derived dietary patterns. This allowed for a more robust exploration of the role of taste perception in habitual food choices and diet quality, and to our knowledge, provided some of the first evidence that taste perception profiles may differentially associate with dietary patterns. The use of a data-driven clustering approach to derive the taste perception profiles in our prior study minimized subjectivity in the analysis, thereby increasing internal validity [24]. However, as this is a statistical study, later mechanistic studies will be required to analyze in greater detail the biological basis of the reported associations. While we could not determine whether taste perception profiles drove food choices in this study, using innovative statistical techniques, significant associations were identified between taste perception profiles and empirical dietary patterns, suggesting important trends to explore in the future. Finally, given the unique study cohort, findings from this work may particularly extend towards high-risk individuals who may benefit most from personalized dietary modification interventions to reduce chronic disease risk. 

## 5. Conclusions

In conclusion, among older adults with metabolic syndrome, we found that taste perception profiles were differentially associated with empirically derived dietary patterns. Participants with high perception of all tastes except umami, generally low, or only high bitter perception tended to follow healthier, prudent-style dietary patterns, though the specific food choices differed by profile. In contrast, participants with high perception of all tastes except bitter tended to follow less healthy, Western-style dietary patterns. These data demonstrate the complexity of taste-diet relations and suggest that integrating taste perception profiles into personalized nutrition efforts may help develop more targeted and specific dietary guidance where more generalized approaches have been less effective. While additional work is needed to translate these initial findings into direct clinical practice, these data support the contention that knowing an individual’s taste perception profile may allow for the development of more effective dietary modification strategies to improve diet quality and reduce chronic disease risk. 

## Figures and Tables

**Table 1:** Selected demographic, clinical, and lifestyle characteristics for the PREDIMED-Plus Valencia cohort, overall and according to taste perception profile ^1^.

		Overall	Taste Perception Profiles ^2^						
			Low All	High Bitter	High Umami	Low Bitter and Umami	High All But Bitter	High All But Umami	*p*
*n* (%)		367	85 (23)	59 (16)	61 (17)	72 (20)	49 (13)	41 (11)	–
Female		202 (55)	36 (42)	30 (51)	33 (54)	42 (58)	34 (69)	27 (66)	0.031
Age (years)		65 ± 4.7	64.5 ± 4.5	65.4 ± 4.9	64.1 ± 4.6	65.2 ± 4.6	66.2 ± 4.9	65.3 ± 4.5	0.217
BMI (kg/m^2^)		32.3 ± 3.6	33.2 ± 3.8	32 ± 3.3	32.4 ± 3.9	32.1 ± 3.5	31.4 ± 3.2	32.4 ± 3.6	0.106
Waist circumference (cm)									
	Females	102 ± 9	103 ± 9	103 ± 9	101 ± 11	104 ± 8	101 ± 8	100 ± 9	0.508
	Males	111 ± 9	113 ± 9	109 ± 7	113 ± 9	109 ± 9	109 ± 7	112 ± 10	0.095
Fasting glucose (mmol/L) ^3^		6.5 ± 1.8	6.5 ± 1.3	6.3 ± 1.6	6.4 ± 1.4	6.5 ± 2.5	7.0 ± 2.2	6.1 ± 1.4	0.241
SBP (mmHg) ^3^		140 ± 17	142 ± 17	139 ± 17	141 ± 19	138 ± 11	140 ± 19	143 ± 18	0.457
DBP (mmHg)		80 ± 9	80 ± 9	80 ± 10	81 ± 8	79 ± 9	80 ± 7	81 ± 10	0.838
Triglycerides (mmol/L) ^3,4^		9.2 ± 4.6	9.2 ± 4.4	9.8 ± 5.0	9.4 ± 4.5	8.9 ± 5.0	9.0 ± 4.6	8.4 ± 4.0	0.383
Total cholesterol (mmol/L) ^3^		11 ± 2.4	11 ± 2.6 ^ac^	11.4 ± 2. 0 ^bc^	11.2 ± 2.4 ^abc^	10.5 ± 2.1 ^ad^	10.3 ± 2.5 ^a^	11.9 ± 2.6 ^b^	0.011
HDL-c (mmol/L) ^3^		2.7 ± 0.6	2.7 ± 0.6	2.7 ± 0.5	2.6 ± 0.6	2.7 ± 0.7	2.8 ± 0.6	2.9 ± 0.5	0.305
LDL-c (mmol/L) ^3^		6.7 ± 2.0	6.6 ± 2.2 ^ab^	7.0 ± 1.7 ^ac^	6.8 ± 2.0 ^ab^	6.2 ± 1.8 ^b^	6.3 ± 1.8 ^bc^	7.3 ± 2.3 ^a^	0.037
Type 2 diabetes		154 (42)	46 (54)	22 (37)	26 (43)	29 (40)	23 (47)	8 (20)	0.011
PA (MET, min/wk)		1798 ± 1665	1661 ± 1522	1645 ± 1471	1733 ± 1966	1804 ± 1466	2330 ± 2104	1753 ± 1419	0.288
Smoking status & history									0.031
	Current/former (<5 yr)	75 (20)	17 (20)	10 (17)	16 (26)	18 (25)	9 (18)	5 (12)	
	Former (>5 yr)	123 (34)	39 (46)	23 (39)	20 (33)	20 (28)	9 (18)	12 (29)	
	Never smoked	169 (46)	29 (34)	26 (44)	25 (41)	34 (47)	31 (63)	24 (59)	
Glucose medications ^5^		118 (32)	32 (38)	20 (34)	20 (33)	24 (33)	17 (35)	5 (12)	0.111
Blood pressure medications		289 (79)	66 (78)	47 (80)	48 (79)	56 (78)	39 (80)	33 (80)	>0.99
Cholesterol medications		240 (65)	62 (73)	35 (59)	37 (61)	49 (68)	31 (63)	26 (63)	0.535

Abbreviations: DBP, diastolic blood pressure; HDL-c, HDL cholesterol; LDL-c, LDL cholesterol; MET, metabolic equivalents; PA, physical activity; SBP, systolic blood pressure; wk, week; yr, year. ^1^ Values are mean ± SD or *n* (%); *N* = 367. Means without a common letter differ significantly; *p* < 0.05. No adjustment was made for multiple comparisons. ^2^ Taste perception profiles were derived via data-driven clustering from taste perception scores; names reflect the defining characteristics of each profile relative to the overall cohort (e.g., Low All had mean perception scores for all 5 tastes roughly 1 SD below the cohort means while High All But Umami had mean perception scores for 4 tastes roughly 1 SD above the cohort means with umami close to the cohort mean). ^3^ 7 values were missing for LDL-c, 4 for HDL-c, 3 for fasting glucose and triglycerides, and 2 for SBP and total cholesterol. ^4^ Triglycerides were log-transformed for normality for statistical comparisons. ^5^ Glucose medications included insulin and Metformin.

**Table 2:** Selected demographic, clinical, and lifestyle characteristics, according to levels of adherence to each empirically derived dietary pattern ^1^.

		Veg/Fruit/WG ^2^				Non-EVOO/Sweet/RG ^2^				Alch/Salt/AnimFat ^2^			
		Low	Moderate	High	*p* ^2^	Low	Moderate	High	*p* ^2^	Low	Moderate	High	*p* ^2^
Dietary pattern score		−1.11 ± 0.60 ^a^	0.06 ± 0.26 ^b^	1.06 ± 0.44 ^c^	<0.001	−1.10 ± 0.49 ^a^	−0.02 ± 0.30 ^b^	1.13 ± 0.42 ^c^	<0.001	−1.06 ± 0.66 ^a^	0.00 ± 0.24 ^b^	1.07 ± 0.50 ^c^	<0.001
Female		49 (40)	68 (56)	85 (70)	0.001	60 (49)	69 (57)	73 (60)	0.202	91 (74)	71 (58)	40 (33)	<0.001
Age (years)		64 ± 5 ^a^	65 ± 5 ^ab^	66 ± 5 ^b^	0.018	65 ± 5	65 ± 4	64 ± 5	0.172	66 ± 5 ^a^	65 ± 4 ^ab^	64 ± 5 ^b^	0.004
BMI (kg/m^2^)		32.6 ± 3.6	32.5 ± 3.8	31.9 ± 3.3	0.206	32.2 ± 3.6	32.1 ± 3.7	32.7 ± 3.5	0.327	32.6 ± 3.8	32.1 ± 3.4	32.3 ± 3.6	0.551
Waist circumference (cm)													
	Females	104 (9)	103 (10)	101 (8)	0.064	102 (8)	101 (9)	104 (9)	0.084	103 (9)	102 (9)	102 (9)	0.533
	Males	111 (9)	113 (9)	109 (8)	0.157	110 (8)	111 (9)	113 (9)	0.426	111 (9)	111 (8)	111 (9)	0.987
Fasting glucose (mmol/L) ^3^		6.6 ± 1.8	6.3 ± 1.5	6.5 ± 2.1	0.516	6.4 ± 2.0	6.5 ± 1.5	6.6 ± 1.8	0.554	6.5 ± 1.9	6.6 ± 2.0	6.4 ± 1.4	0.662
SBP (mmHg) ^3^		141.9 ± 18	140.8 ± 16	138.2 ± 17	0.209	142 (16)	142 (17)	137 (17)	0.054	140 (18)	141 (15)	140 (17)	0.922
DBP (mmHg)		81.3 ± 10	79 ± 10	79 ± 7	0.142	81 (9)	80 (9)	79 (9)	0.398	79 (9) ^a^	79 (9) ^ab^	82 (9) ^b^	0.013
Triglycerides (mmol/L) ^3,4^		10.0 ± 5.3	8.9 ± 3.9	8.6 ± 4.4	0.066	8.7 ± 3.9	9.5 ± 4.8	9.4 ± 5.0	0.399	8.5 ± 3.2	8.9 ± 4.2	10.2 ± 5.9	0.123
Total cholesterol (mmol/L) ^3^		11.0 ± 2.6	10.7 ± 2.2	11.4 ± 2.3	0.065	10.9 ± 2.5	11.4 ± 2.4	10.8 ± 2.3	0.119	10.9 ± 2.2	10.7 ± 2.5	11.4 ± 2.5	0.047
HDL-C (mmol/L) ^3^		2.7 ± 0.6	2.7 ± 0.6	2.8 ± 0.6	0.460	2.8 ± 0.6	2.6 ± 0.6	2.7 ± 0.7	0.105	2.7 ± 0.6	2.7 ± 0.6	2.7 ± 0.6	0.917
LDL-C (mmol/L) ^3^		6.6 ± 2.1 ^ab^	6.4 ± 1.9 ^a^	7.0 ± 2.0 ^b^	0.038	6.5 ± 2.0	7.0 ± 2.1	6.5 ± 1.8	0.097	6.6 ± 2.0	6.5 ± 2.0	6.9 ± 2.1	0.249
Diabetes		51 (41)	51 (42)	52 (43)	0.982	39 (32)	52 (43)	63 (52)	0.007	50 (41)	56 (46)	48 (39)	0.547
Energy intake (kcal/d)		2371 (561)	2418 (535)	2392 (472)	0.776	2425 (539)	2301 (492)	2455 (528)	0.052	2419 (572)	2314 (473)	2448 (513)	0.111
PA (MET, min/wk)		1836(2035)	1795 (1417)	1763 (1484)	0.943	2165 (1829) ^a^	1778 (1454) ^ab^	1447 (1624) ^b^	0.003	1741 (1797)	1920 (1527)	1734 (1666)	0.615
Smoking status & history					0.012				0.528				<0.001
	Current/former (<5 yr)	36 (29)	17 (14)	22 (18)		26 (21)	20 (16)	29 (24)		20 (16)	20 (16)	35 (29)	
	Former (>5 yr)	43 (35)	44 (36)	36 (30)		45 (37)	41 (34)	37 (30)		27 (22)	47 (39)	49 (40)	
	Never smoked	44 (36)	61 (50)	64 (52)		52 (42)	61 (50)	56 (46)		76 (62)	55 (45)	38 (31)	
Glucose medications ^5^		40 (33)	38 (31)	40 (33)	0.958	31 (25)	35 (29)	52 (43)	0.009	37 (30)	45 (37)	36 (30)	0.390
Blood pressure medications		99 (80)	92 (75)	98 (80)	0.544	100 (81)	92 (75)	97 (80)	0.513	99 (80)	96 (79)	94 (77)	0.805
Cholesterol medications		82 (67)	84 (69)	74 (61)	0.378	77 (63)	81 (66)	82 (67)	0.720	70 (57)	87 (71)	83 (68)	0.046

Abbreviations: DBP, diastolic blood pressure; HDL-c, HDL cholesterol; LDL-c, LDL cholesterol; MET, metabolic equivalents; PA, physical activity; SBP, systolic blood pressure; wk, week; yr, year. ^1^ Values are mean ± SD or *n* (%); *N* = 367. Means without a common letter differ significantly; *p* < 0.05. ^2^ Cut-offs for low, moderate, and high adherence were determined based on tertials; tertial 1, low (*n* = 123); tertial 2, moderate (*n* = 122); tertial 3, high (*n* = 122). ^3^ 7 values were missing for LDL-c, 4 for HDL-c, 3 for fasting glucose and triglycerides, 2 for SBP and total cholesterol. ^4^ Triglycerides were log-transformed for normality for statistical comparisons. ^5^ Glucose medications included insulin and Metformin.

## Data Availability

Data described in the manuscript are not expected to be made available outside the core research group, as neither the participants’ consent forms nor ethics approval included permission for open access. However, we follow a controlled data-sharing collaboration model, and code book, analytic code, and data for collaborations will be available upon request pending application and approval. Investigators who are interested in this study can contact the corresponding author.

## Supplementary Materials

The following supporting information can be downloaded at: https://www.mdpi.com/article/10.3390/nu14010142/s1, Figure S1: Flowchart of PREDIMED-Plus Valencia participants, Figure S2: Scree plot of the eigenvalues used to determine the number of principal components to retain, Table S1: Food groups and food items for dietary pattern analysis, Table S2: Rotated factor loadings for all food groups from principal component analysis, Table S3: ORs and corresponding 95% CIs of moderate or high relative to low adherence to the Veg/Fruit/WG dietary pattern among participants with each taste perception profile, Table S4: ORs and corresponding 95% CIs of moderate or high relative to low adherence to the Non-EVOO/Sweet/RG dietary pattern among participants with each taste perception profile, Table S5: ORs and corresponding 95% CIs of moderate or high relative to low adherence to the Alch/Salt/AnimFat dietary pattern among participants with each taste perception profile.

## References

[1] Van Horn L., Cornelis M.C. (2019). US Dietary Guidance—Is It Working?. JAMA.

[2] The US Burden of Disease Collaborators (2018). The State of US Health, 1990–2016: Burden of Diseases, Injuries, and Risk Factors Among US States. JAMA.

[3] Afshin A., Sur P.J., Fay K.A., Cornaby L., Ferrara G., Salama J.S., Mullany E.C., Abate K.H., Abbafati C., Abebe Z. (2019). Health Effects of Dietary Risks in 195 Countries, 1990–2017: A Systematic Analysis for the Global Burden of Disease Study 2017. Lancet.

[4] U.S. Department of Agriculture and U.S. Department of Health and Human Services (2020). Dietary Guidelines for Americans, 2020–2025.

[5] Ordovas J.M., Ferguson L.R., Tai E.S., Mathers J.C. (2018). Personalised Nutrition and Health. BMJ.

[6] Bush C.L., Blumberg J.B., El-Sohemy A., Minich D.M., Ordovás J.M., Reed D.G., Behm V.A.Y. (2020). Toward the Definition of Personalized Nutrition: A Proposal by The American Nutrition Association. J. Am. Coll. Nutr..

[7] Jinnette R., Narita A., Manning B., McNaughton S.A., Mathers J.C., Livingstone K.M. (2021). Does Personalized Nutrition Advice Improve Dietary Intake in Healthy Adults? A Systematic Review of Randomized Controlled Trials. Adv. Nutr..

[8] Zeevi D., Korem T., Zmora N., Israeli D., Rothschild D., Weinberger A., Ben-Yacov O., Lador D., Avnit-Sagi T., Lotan-Pompan M. (2015). Personalized Nutrition by Prediction of Glycemic Responses. Cell.

[9] Celis-Morales C., Livingstone K.M., Marsaux C.F.M., Macready A.L., Fallaize R., O’Donovan C.B., Woolhead C., Forster H., Walsh M.C., Navas-Carretero S. (2016). Effect of Personalized Nutrition on Health-Related Behaviour Change: Evidence from the Food4me European Randomized Controlled Trial. Int. J. Epidemiol..

[10] Horne J., Madill J., O’Connor C., Shelley J., Gilliland J. (2018). A Systematic Review of Genetic Testing and Lifestyle Behaviour Change: Are We Using High-Quality Genetic Interventions and Considering Behaviour Change Theory?. Lifestyle Genom..

[11] Biesiekierski J.R., Livingstone K.M., Moschonis G. (2019). Personalised Nutrition: Updates, Gaps and Next Steps. Nutrients.

[12] Breslin P.A.S. (2013). An Evolutionary Perspective on Food Review and Human Taste. Curr. Biol..

[13] Hayes J.E., Feeney E.L., Allen A.L. (2013). Do Polymorphisms in Chemosensory Genes Matter for Human Ingestive Behavior?. Food Qual. Prefer..

[14] Shen Y., Kennedy O.B., Methven L. (2016). Exploring the Effects of Genotypical and Phenotypical Variations in Bitter Taste Sensitivity on Perception, Liking and Intake of Brassica Vegetables in the UK. Food Qual. Prefer..

[15] Duffy V.B., Hayes J.E., Davidson A.C., Kidd J.R., Kidd K.K., Bartoshuk L.M. (2010). Vegetable Intake in College-Aged Adults Is Explained by Oral Sensory Phenotypes and TAS2R38 Genotype. Chem. Percept..

[16] Dinehart M.E., Hayes J.E., Bartoshuk L.M., Lanier S.L., Duffy V.B. (2006). Bitter Taste Markers Explain Variability in Vegetable Sweetness, Bitterness, and Intake. Physiol. Behav..

[17] Shafaie Y., Koelliker Y., Hoffman D.J., Tepper B.J. (2013). Energy Intake and Diet Selection during Buffet Consumption in Women Classified by the 6-n-Propylthiouracil Bitter Taste Phenotype. Am. J. Clin. Nutr..

[18] Cattaneo C., Riso P., Laureati M., Gargari G., Pagliarini E. (2019). Exploring Associations between Interindividual Differences in Taste Perception, Oral Microbiota Composition, and Reported Food Intake. Nutrients.

[19] Jayasinghe S.N., Kruger R., Walsh D.C., Cao G., Rivers S., Richter M., Breier B.H. (2017). Is Sweet Taste Perception Associated with Sweet Food Liking and Intake?. Nutrients.

[20] Veček N.N., Mucalo L., Dragun R., Miličević T., Pribisalić A., Patarčić I., Hayward C., Polašek O., Kolčić I. (2020). The Association between Salt Taste Perception, Mediterranean Diet and Metabolic Syndrome: A Cross-Sectional Study. Nutrients.

[21] Ong J.-S., Hwang L.-D., Zhong V.W., An J., Gharahkhani P., Breslin P.A.S., Wright M.J., Lawlor D.A., Whitfield J., MacGregor S. (2018). Understanding the Role of Bitter Taste Perception in Coffee, Tea and Alcohol Consumption through Mendelian Randomization. Sci. Rep..

[22] Hu F.B. (2002). Dietary Pattern Analysis: A New Direction in Nutritional Epidemiology. Curr. Opin. Lipidol..

[23] Ocké M.C. (2013). Evaluation of Methodologies for Assessing the Overall Diet: Dietary Quality Scores and Dietary Pattern Analysis. Proc. Nutr. Soc..

[24] Gervis J.E., Chui K.K.H., Ma J., Coltell O., Fernández-Carrión R., Sorlí J.V., Barragán R., Fitó M., González J.I., Corella D. (2021). Data-Driven Clustering Approach to Derive Taste Perception Profiles from Sweet, Salt, Sour, Bitter, and Umami Perception Scores: An Illustration among Older Adults with Metabolic Syndrome. J. Nutr..

[25] Tepper B.J. (2021). Toward a Better Understanding of Diet–Taste Relations. J. Nutr..

[26] Martínez-González M.A., Buil-Cosiales P., Corella D., Bulló M., Fitó M., Vioque J., Romaguera D., Martínez J.A., Wärnberg J., López-Miranda J. (2019). Cohort Profile: Design and Methods of the PREDIMED-Plus Randomized Trial. Int. J. Epidemiol..

[27] Alberti K.G.M.M., Eckel R.H., Grundy S.M., Zimmet P.Z., Cleeman J.I., Donato K.A., Fruchart J.-C., James W.P.T., Loria C.M., Smith S.C. (2009). Harmonizing the Metabolic Syndrome. Circulation.

[28] Coltell O., Sorlí J.V., Asensio E.M., Fernández-Carrión R., Barragán R., Ortega-Azorín C., Estruch R., González J.I., Salas-Salvadó J., Lamon-Fava S. (2019). Association between Taste Perception and Adiposity in Overweight or Obese Older Subjects with Metabolic Syndrome and Identification of Novel Taste-Related Genes. Am. J. Clin. Nutr..

[29] Willett W. (2012). Implications of Total Energy Intake for Epidemiologic Analyses. Nutritional Epidemiology.

[30] Rosique-Esteban N., Díaz-López A., Martínez-González M.A., Corella D., Goday A., Martínez J.A., Romaguera D., Vioque J., Arós F., Garcia-Rios A. (2017). Leisure-Time Physical Activity, Sedentary Behaviors, Sleep, and Cardiometabolic Risk Factors at Baseline in the PREDIMED-PLUS Intervention Trial: A Cross-Sectional Analysis. PLoS ONE.

[31] Fernández-Ballart J.D., Piñol J.L., Zazpe I., Corella D., Carrasco P., Toledo E., Perez-Bauer M., Martínez-González M.Á., Salas-Salvadó J., Martín-Moreno J.M. (2010). Relative Validity of a Semi-Quantitative Food-Frequency Questionnaire in an Elderly Mediterranean Population of Spain. Br. J. Nutr..

[32] Willett W.C., Howe G.R., Kushi L.H. (1997). Adjustment for Total Energy Intake in Epidemiologic Studies. Am. J. Clin. Nutr..

[33] Schwedhelm C., Iqbal K., Knüppel S., Schwingshackl L., Boeing H. (2018). Contribution to the Understanding of How Principal Component Analysis–Derived Dietary Patterns Emerge from Habitual Data on Food Consumption. Am. J. Clin. Nutr..

[34] Thorpe M.G., Milte C.M., Crawford D., McNaughton S.A. (2016). A Comparison of the Dietary Patterns Derived by Principal Component Analysis and Cluster Analysis in Older Australians. Int. J. Behav. Nutr. Phys. Act..

[35] Schulze M.B., Hoffmann K., Kroke A., Boeing H. (2003). An Approach to Construct Simplified Measures of Dietary Patterns from Exploratory Factor Analysis. Br. J. Nutr..

[36] Molina L., Sarmiento M., Peñafiel J., Donaire D., Garcia-Aymerich J., Gomez M., Ble M., Ruiz S., Frances A., Schröder H. (2017). Validation of the Regicor Short Physical Activity Questionnaire for the Adult Population. PLoS ONE.

[37] Fung T.T., Rimm E.B., Spiegelman D., Rifai N., Tofler G.H., Willett W.C., Hu F.B. (2001). Association between Dietary Patterns and Plasma Biomarkers of Obesity and Cardiovascular Disease Risk. Am. J. Clin. Nutr..

[38] Aranceta-Bartrina J., Partearroyo T., López-Sobaler A.M., Ortega R.M., Varela-Moreiras G., Serra-Majem L., Pérez-Rodrigo C., Collaborative Group for the Dietary Guidelines for the Spanish Population (SENC) (2019). Updating the Food-Based Dietary Guidelines for the Spanish Population: The Spanish Society of Community Nutrition (SENC) Proposal. Nutrients.

[39] Puputti S., Hoppu U., Sandell M. (2019). Taste Sensitivity Is Associated with Food Consumption Behavior but Not with Recalled Pleasantness. Foods.

[40] Sjöstrand A.E., Sjödin P., Hegay T., Nikolaeva A., Shayimkulov F., Blum M.G., Heyer E., Jakobsson M. (2020). Taste Perception and Lifestyle: Insights from Phenotype and Genome Data among Africans and Asians. Eur. J. Hum. Genet..

[41] Barragán R., Coltell O., Portolés O., Asensio E.M., Sorlí J.V., Ortega-Azorín C., González J.I., Sáiz C., Fernández-Carrión R., Ordovas J.M. (2018). Bitter, Sweet, Salty, Sour and Umami Taste Perception Decreases with Age: Sex-Specific Analysis, Modulation by Genetic Variants and Taste-Preference Associations in 18 to 80 Year-Old Subjects. Nutrients.

[42] Martinez-Perez C., San-Cristobal R., Guallar-Castillon P., Martínez-González M.Á., Salas-Salvadó J., Corella D., Castañer O., Martinez J.A., Alonso-Gómez Á.M., Wärnberg J. (2021). Use of Different Food Classification Systems to Assess the Association between Ultra-Processed Food Consumption and Cardiometabolic Health in an Elderly Population with Metabolic Syndrome (PREDIMED-Plus Cohort). Nutrients.

[43] Blondin S.A., Mueller M.P., Bakun P.J., Choumenkovitch S.F., Tucker K.L., Economos C.D. (2016). Cross-Sectional Associations between Empirically-Derived Dietary Patterns and Indicators of Disease Risk among University Students. Nutrients.

[44] Northstone K., Ness A., Emmett P., Rogers I. (2008). Adjusting for Energy Intake in Dietary Pattern Investigations Using Principal Components Analysis. Eur. J. Clin. Nutr..

[45] Markussen M.S., Veierød M.B., Ursin G., Andersen L.F. (2016). The Effect of Under-Reporting of Energy Intake on Dietary Patterns and on the Associations between Dietary Patterns and Self-Reported Chronic Disease in Women Aged 50–69 Years. Br. J. Nutr..

[46] Low J.Y.Q., Lacy K.E., McBride R., Keast R.S.J. (2016). The Association between Sweet Taste Function, Anthropometry, and Dietary Intake in Adults. Nutrients.

[47] Mattes R.D. (1988). Reliability of Psychophysical Measures of Gustatory Function. Percept. Psychophys..

[48] Griep M., Borg E., Collys K., Massart D. (1998). Category Ratio Scale as an Alternative to Magnitude Matching for Age-Related Taste and Odour Perception. Food Qual. Prefer..

[49] Genick U.K., Kutalik Z., Ledda M., Destito M.C., Souza M.M., Cirillo C.A., Godinot N., Martin N., Morya E., Sameshima K. (2011). Sensitivity of genome-wide-association signals to phenotyping strategy: The PROP-TAS2R38 taste association as a benchmark. PLoS ONE.

[50] Ledda M., Kutalik Z., Souza Destito M.C., Souza M.M., Cirillo C.A., Zamboni A., Martin N., Morya E., Sameshima K., Beckmann J.S. (2014). GWAS of human bitter taste perception identifies new loci and reveals additional complexity of bitter taste genetics. Hum. Mol. Genet..

[51] Bartoshuk L.M., Duffy V.B., Miller I.J. (1994). PTC/PROP Tasting: Anatomy, Psychophysics, and Sex Effects. Physiol. Behav..

[52] Duffy V.B., Davidson A.C., Kidd J.R., Kidd K.K., Speed W.C., Pakstis A.J., Reed D.R., Snyder D.J., Bartoshuk L.M. (2004). Bitter Receptor Gene (TAS2R38), 6-n-propylthiouracil (PROP) Bitterness and Alcohol Intake. Alcohol. Clin. Exp. Res..

[53] Hansen J.L., Reed D.R., Wright M.J., Martin N.G., Breslin P.A.S. (2006). Heritability and Genetic Covariation of Sensitivity to PROP, SOA, Quinine HCl, and Caffeine. Chem. Senses.

[54] Hayes J.E., Bartoshuk L.M., Kidd J.R., Duffy V.B. (2008). Supertasting and PROP Bitterness Depends on More than the TAS2R38 Gene. Chem. Senses.

[55] Running C.A., Craig B.A., Mattes R.D. (2015). Oleogustus: The Unique Taste of Fat. Chem. Senses.

[56] Keast R.S., Costanzo A. (2015). Is Fat the Sixth Taste Primary? Evidence and Implications. Flavour.

