# Supplementary material for: Associations between Taste Perception Profiles and Empirically Derived Dietary Patterns: An Exploratory Analysis among Older Adults with Metabolic Syndrome
Source: Nutrients. 2021 Dec 29;14(1):142. doi: 10.3390/nu14010142 (PMC8747383; doi:10.3390/nu14010142)
Supplement: Supplementary file 1 [file nutrients-14-00142-s001.zip › nutrients-1463880-supplementary.pdf]

ONLINE SUPPORTING MATERIAL

Associations between taste perception profiles and empirically derived dietary patterns: An exploratory analysis among older adults with metabolic syndrome

Julie E. Gervis, Rebeca Fernández-Carrión, Kenneth K. H. Chui, Jiantao Ma, Oscar Coltell, Jose V. Sorli, Eva M. Asensio, Carolina Ortega-Azorín, José Alejandro Pérez-Fidalgo, Olga Portolés, Alice H. Lichtenstein[†], Dolores Corella[†]

[†] These authors contributed equally as senior authors

Table of Contents

Supplementary Figure S1. Flowchart of PREDIMED-Plus Valencia participants.

Supplementary Figure S2. Scree plot of the eigenvalues used to determine the number of principal components to retain.

Supplementary Table S1. Food groups and food items for dietary pattern analysis.

Supplementary Table S2. Rotated factor loadings for all food groups from Principal Component Analysis.

Supplementary Table S3. ORs and corresponding 95% CIs of moderate or high relative to low adherence to the Veg/Fruit/WG dietary pattern among participants with each taste perception profile.

Supplementary Table S4. ORs and corresponding 95% CIs of moderate or high relative to low adherence to the Non-EVOO/Sweet/RG dietary pattern among participants with each taste perception profile.

Supplementary Table S5. ORs and corresponding 95% CIs of moderate or high relative to low adherence to the Alch/Salt/AnimFat dietary pattern among participants with each taste perception profile.

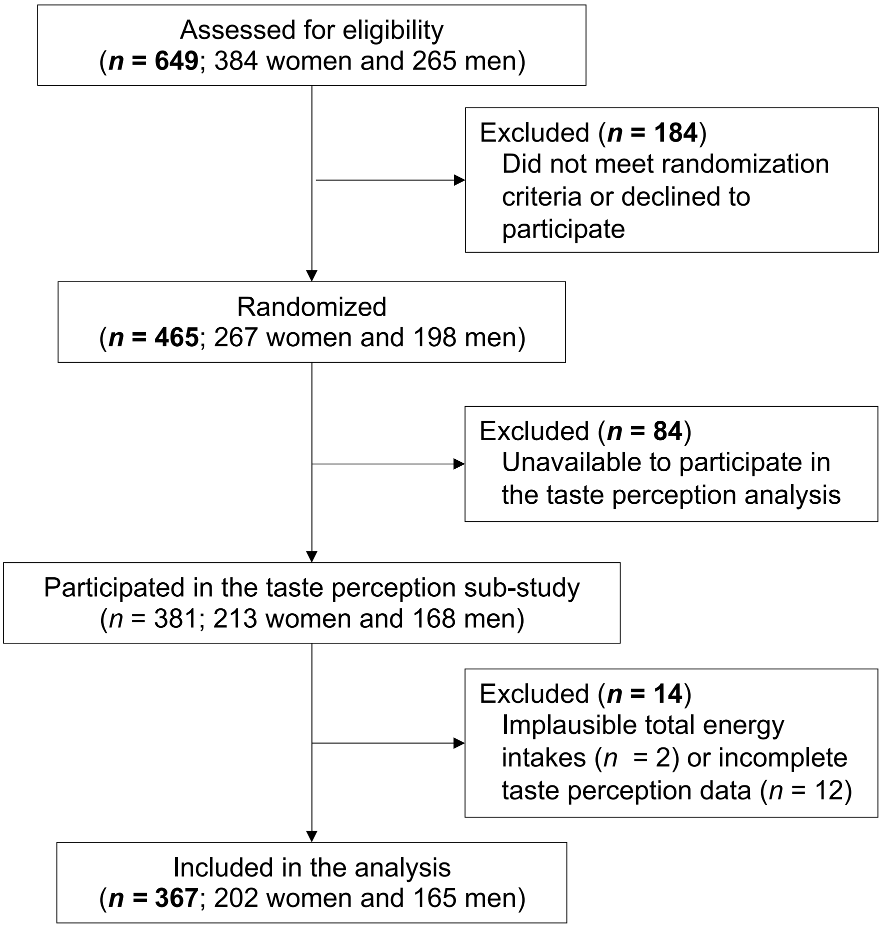

Supplementary Figure S1. Flowchart of PREDIMED-Plus Valencia participants.

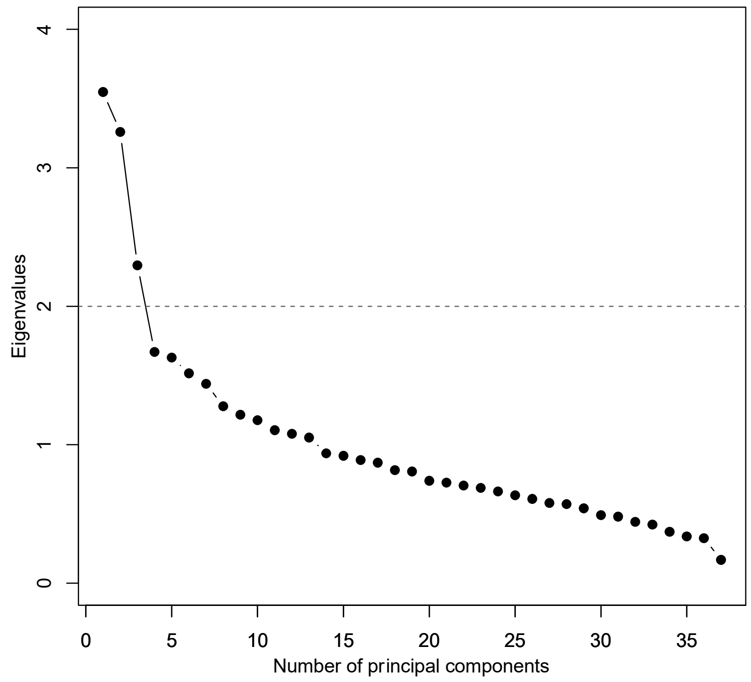

Supplementary Figure S2. Scree plot of the eigenvalues used to determine the number of principal components to retain. Ultimately, three principal components were retained based on their eigenvalues >2.

Supplementary Table S1. Food groups and food items for dietary pattern analysis.

Food groups	Food items
Fresh fish	White fish; blue fish
Shellfish	Canned natural fish; fish in oil; salted fish
Canned & salted fish	Oysters, clams, mussels; squid, octopus, cuttlefish; prawns, crayfish
Poultry	Chicken with/without skin
Red meat	Beef, pork; lamb; rabbit; liver; hamburgers
Processed meat	Serrano ham; cooked ham; sausages; pates; bacon
Eggs	
Low & no-fat dairy	Nonfat yogurt; low-fat milk; skim milk
Full-fat dairy	Full-fat yogurt; whole milk; sweet cream; cheese, cheese curds; spreads; condensed milk
Cruciferous vegetables	Cabbage, cauliflower, broccoli
Leafy green vegetables	Chard, spinach; lettuce, endive
Red & orange vegetables	Tomatoes; carrots, pumpkin
Other vegetables	Peppers; asparagus; green beans; onions; mushrooms; zucchini, eggplant; cucumber; gazpacho
Citrus fruits	Oranges, tangerines, and grapefruits
Non-citrus fruits	Strawberries; watermelon; melons; kiwi; bananas; apples, pears; cherries, plums; peaches, apricots, nectarines; grapes; dried fruits; fruit cups (in syrup)
Potatoes	Roasted and cooked potatoes; homemade fried potatoes; commercial fried potatoes
Legumes	Lentils; dried beans; chickpeas; peas and beans
Nuts	Almonds; pistachios; walnuts; other nuts
Whole grains	Whole grain bread; whole grain pasta; whole grain fiber biscuits; brown rice; muesli, oats
Refined grains	White bread; white rice; white spaghetti or macaroni; breakfast cereals
EVOO	
Refined olive oil	Refined olive oil; pomace olive oil
Vegetable oil	Corn oil; sunflower oil; soy oil; mixture of vegetable oils; margarines
Chocolate & dairy desserts	Chocolate biscuits; cocoa powder; chocolates and bonbons; custard, pudding; ice cream
Biscuits, pastries & Cakes	Biscuits; homemade pastries; commercial pastries; donuts; cupcakes; muffins; cake; turron; shortbread, marzipan; pastries
Olives	Olives
Tomato sauces & mustard	Fried tomato sauce (sofrito) and ketchup; mustard
Jelly, sugar & honey	
Butter & mayo	Butter; mayonnaise
Salty snacks	Other snacks besides fried potatoes (e.g., chips, pretzels)
Croquettes	Croquettes, fritters, and dumplings
Soups & cream packets	
Pizza	
Coffee	Decaf coffee; caffeinated coffee
Tea	
SSBs	Sodas with sugar; bottled fruit juice; non-alcoholic wine (e.g., musts)
Natural fruit juices	Orange juice; natural fruit juice
Wine	Rosé; sweet wine; young red wine; aged red wine; white wine; champagne
Beer	
Spirits	Liqueurs, anise; whiskey, gin, vodka

EVOO, extra virgin olive oil; SSBs, sugar-sweetened beverages

Supplementary Table S2. Rotated factor loadings for all food groups from Principal Component Analysis[1]

Veg/Fruit/WG		Non-EVOO/Sweet/RG		Alch/Salt/AnimFat	
Other veg	0.66*	Refined olive oil	0.73*	Wine	0.55*
Leafy green veg	0.63*	Vegetable oil	0.47*	Olives	0.54*
Red & orange veg	0.62*	Biscuits, pastries & cakes	0.44*	Beer	0.52*
Non-citrus fruit	0.61*	Refined grains	0.40*	Spirits	0.50*
Whole grains	0.45*	Croquettes	0.30*	Eggs	0.40*
Canned/salted fish	0.43*	Butter & mayo	0.27*	Shellfish	0.36*
Cruciferous veg	0.32*	Chocolate & dairy desserts	0.27*	Canned/salted fish	0.33*
Low-fat dairy	0.30*	SSBs	0.25*	Red meat	0.27*
Tea	0.27*	Canned/salted fish	0.22*	Processed meat	0.25*
Poultry	0.24*	Tomato sauces & mustard	0.19	Butter & mayo	0.22*
Fresh fish	0.22*	Olives	0.15	Other veg	0.19
Shellfish	0.18	Soup & cream packets	0.11	Pizza	0.17
Refined olive oil	0.18	Poultry	0.10	Potatoes	0.16
Natural fruit juices	0.18	Low-fat dairy	0.10	Tomato sauces & mustard	0.16
Potatoes	0.15	Non-citrus fruit	0.09	Poultry	0.14
Nuts	0.14	Pizza	0.09	Red & orange veg	0.13
Chocolate & dairy desserts	0.11	Potatoes	0.08	Fresh fish	0.13
Citrus fruits	0.09	Coffee	0.05	Chocolate & dairy desserts	0.12
Eggs	0.07	Eggs	0.04	Croquettes	0.12
Pizza	0.06	Red meat	0.02	Coffee	0.12
SSBs	0.04	Full-fat dairy	0.01	Refined grains	0.06
EVOO	0.03	Shellfish	0.00	Full-fat dairy	0.05
Red meat	0.02	Other veg	-0.02	SSBs	0.05
Legumes	0.02	Citrus fruits	-0.03	Salty snacks	0.05
Coffee	0.00	Beer	-0.03	Leafy green veg	0.04
Olives	-0.01	Cruciferous veg	-0.06	EVOO	0.04
Jelly, sugar & honey	-0.04	Red & orange veg	-0.06	Jelly, sugar & honey	0.02
Processed meat	-0.05	Salty snacks	-0.06	Refined olive oil	0.01
Vegetable oil	-0.05	Tea	-0.07	Natural fruit juices	0.00
Biscuits, pastries & cakes	-0.09	Leafy green veg	-0.09	Biscuits, pastries & cakes	-0.02
Tomato sauces & mustard	-0.13	Spirits	-0.12	Tea	-0.02
Butter & mayo	-0.13	Processed meat	-0.14	Soup & cream packets	-0.07
Wine	-0.13	Jelly, sugar & honey	-0.17	Cruciferous veg	-0.09
Soup & cream packets	-0.14	Natural fruit juices	-0.19	Legumes	-0.15
Salty snacks	-0.13	Wine	-0.29*	Vegetable oil	-0.15
Full-fat dairy	-0.20	Whole grains	-0.31*	Citrus fruits	-0.16
Spirits	-0.25*	Legumes	-0.33*	Nuts	-0.18
Beer	-0.25*	Fresh fish	-0.33*	Non-citrus fruit	-0.20
Croquettes	-0.30*	Nuts	-0.37*	Whole grains	-0.23*
Refined grains	-0.47*	EVOO	-0.73*	Low-fat dairy	-0.25*

[1]Values are rotated factor loadings from PCA, arranged in descending order from higher factor loading (highest consumption) to lowest factor loading (lowest consumption). Food groups with a factor loading >|0.20| were considered to contribute significantly to a respective dietary pattern and were marked with an asterisks (*). Alch/Salt/AnimFat, Alcohol, Salty Foods, and Animal Fats dietary pattern; EVOO, extra virgin olive oil; Non-EVOO/Sweet/RG, Non-EVOO, Sweets, and Refined Grains dietary pattern; SSBs, sugar-sweetened beverages; Veg/Fruit/WG, Vegetables, Fruits, and Whole Grains dietary pattern.

Supplementary Table S3. ORs and corresponding 95% CIs of moderate or high relative to low adherence to the Veg/Fruit/WG dietary pattern among participants with each taste perception profile.

	Reference taste perception profiles												
	Low All		High Bitter		High Umami		Low Bitter and Umami		High All But Bitter		High All But Umami		*p*[3]
	OR (95% CI)	*p*[2]	OR (95% CI)	*p*[2]	OR (95% CI)	*p*[2]	OR (95% CI)	*p*[2]	OR (95% CI)	*p*[2]	OR (95% CI)	*p*[2]	
Low All													
Moderate	1.00	–											
High	1.00	–											
High Bitter													
Moderate	0.87 (0.66, 1.15)	0.327	1.00	–									
High	1.37 (0.99, 1.90)	0.056	1.00	–									
High Umami													
Moderate	1.13 (0.85, 1.51)	0.327	1.30 (0.97, 1.73)	0.075	1.00	–							
High	1.27 (0.93, 1.71)	0.128	0.92 (0.68, 1.25)	0.602	1.00	–							
Low Bitter & Umami													
Moderate	1.93 (1.44, 2.57)	0.402	2.21 (1.66, 2.95)	<0.001	1.70 (1.28, 2.27)	<0.001	1.00	–					
High	1.16 (0.91, 1.49)	0.229	0.85 (0.66, 1.09)	0.196	0.92 (0.72, 1.18)	0.514	1.00	–					
High All But Bitter													
Moderate	1.17 (0.86, 1.59)	<0.001	1.34 (0.99, 1.82)	0.061	1.03 (0.76, 1.40)	0.836	0.61 (0.45, 0.83)	0.002	1.00	–			
High	1.13 (0.82, 1.54)	0.460	0.82 (0.60, 1.12)	0.212	0.89 (0.65, 1.22)	0.463	0.97 (0.70, 1.33)	0.830	1.00	–			
High All But Umami													
Moderate	1.60 (1.18, 2.18)	0.320	1.84 (1.34, 2.53)	<0.001	1.42 (1.03, 1.94)	0.031	0.83 (0.60, 1.14)	0.255	1.37 (1.00, 1.88)	0.470	1.00	–	
High	1.99 (1.45, 2.74)	<0.001	1.45 (1.05, 2.00)	0.023	1.57 (1.14, 2.17)	0.006	1.71 (1.23, 2.37)	0.001	1.77 (1.28, 2.44)	<0.001	1.00	–	
Global *p*-value[3]													0.631

[1]Values are ORs (95% CI) estimated via multinomial logistic regression, with adjustment for age, sex, physical activity, smoking status, use of antihypertensive and cholesterol lowering medication, type 2 diabetes, and BMI. Veg/Fruit/WG, Vegetables, Fruits, and Whole Grains dietary pattern.

[2]*p*-values from Chi Square tests for differences from the reference taste perception profile (calculated by column).

[3]*p*-value from log likelihood ratio test of joint significance in fully adjusted multinomial logistic regression models. Only 1 *p*-value is provided, as it is identical for all models regardless of the reference profile.

Supplementary Table S4. ORs and corresponding 95% CIs of moderate or high relative to low adherence to the Non-EVOO/Sweet/RG dietary pattern among participants with each taste perception profile.

	Reference taste perception profiles												
	Low All		High Bitter		High Umami		Low Bitter and Umami		High All But Bitter		High All But Umami		*p*[3]
	OR (95% CI)	*p*[2]	OR (95% CI)	*p*[2]	OR (95% CI)	*p*[2]	OR (95% CI)	*p*[2]	OR (95% CI)	*p*[2]	OR (95% CI)	*p*[2]	
Low All													
Moderate	1.00	–											
High	1.00	–											
High Bitter													
Moderate	0.91 (0.67, 1.22)	0.521	1.00	–									
High	1.24 (0.90, 1.71)	0.182	1.00	–									
High Umami													
Moderate	2.31 (1.72, 3.10)	<0.001	2.54 (1.90, 3.39)	<0.001	1.00	–							
High	1.70 (1.30, 2.22)	<0.001	1.37 (1.05, 1.78)	0.019	1.00	–							
Low Bitter & Umami													
Moderate	1.77 (1.34, 2.34)	<0.001	1.95 (1.48, 2.58)	<0.001	0.77 (0.58, 1.02)	0.066	1.00	–					
High	1.75 (1.34, 2.27)	<0.001	1.41 (1.08, 1.82)	0.010	1.03 (0.79, 1.34)	0.844	1.00	–					
High All But Bitter													
Moderate	1.09 (0.80, 1.46)	0.590	1.20 (0.88, 1.62)	0.246	0.47 (0.35, 0.63)	<0.001	0.61 (0.45, 0.83)	0.002	1.00	–			
High	1.81 (1.29, 2.53)	<0.001	1.45 (1.04, 2.03)	0.028	1.06 (0.75, 1.49)	0.729	1.03 (0.74, 1.45)	0.844	1.00	–			
High All But Umami													
Moderate	2.31 (1.62, 3.29)	<0.001	2.54 (1.78, 3.63)	<0.001	1.00 (0.70, 1.43)	0.998	1.30 (0.91, 1.86)	0.149	2.12 (1.49, 3.03)	<0.001	1.00	–	
High	1.52 (1.14, 2.02)	0.005	1.22 (0.91, 1.63)	0.178	0.89 (0.66, 1.20)	0.450	0.87 (0.65, 1.17)	0.349	0.84 (0.63, 1.12)	0.238	1.00	–	
Global *p*-value[3]													0.372

[1]Values are ORs (95% CI) estimated via multinomial logistic regression, with adjustment for age, sex, physical activity, smoking status, use of antihypertensive and cholesterol lowering medication, type 2 diabetes, and BMI. EVOO, Extra virgin olive oil; Non-EVOO/Sweet/RG, Non-EVOO, Sweets, and Refined Grains dietary pattern.

[2]*p*-values from Chi Square tests for differences from the reference taste perception profile (calculated by column).

[3]*p*-value from log likelihood ratio test of joint significance in fully adjusted multinomial logistic regression models. Only 1 *p*-value is provided, as it is identical for all models regardless of the reference profile.

Supplementary Table S5. ORs and corresponding 95% CIs of moderate or high relative to low adherence to the Alch/Salt/AnimFat dietary pattern among participants with each taste perception profile.

	Reference taste perception profiles												
	Low All		High Bitter		High Umami		Low Bitter & Umami		High All But Bitter		High All But Umami		p^3
	OR (95% CI)	p^2	OR (95% CI)	p^2	OR (95% CI)	p^2	OR (95% CI)	p^2	OR (95% CI)	p^2	OR (95% CI)	p^2	
Low All													
Moderate	1.00	–											
High	1.00	–											
High Bitter													
Moderate	0.43 (0.33, 0.57)	<0.001	1.00	–									
High	1.18 (0.83, 1.68)	0.360	1.00	–									
High Umami													
Moderate	1.23 (0.90, 1.66)	0.189	2.84 (2.11, 3.81)	<0.001	1.00	–							
High	1.17 (0.90, 1.52)	0.242	0.99 (0.77, 1.28)	0.953	1.00	–							
Low Bitter & Umami													
Moderate	0.73 (0.53, 1.01)	0.057	1.69 (1.23, 2.31)	0.001	0.60 (0.43, 0.82)	0.001	1.00	–					
High	0.71 (0.56, 0.92)	0.009	0.61 (0.48, 0.77)	<0.001	0.61 (0.48, 0.78)	<0.001	1.00	–					
High All But Bitter													
Moderate	0.68 (0.49, 0.95)	0.024	1.57 (1.12, 2.21)	0.009	0.55 (0.40, 0.77)	<0.001	0.93 (0.67, 1.30)	0.681	1.00	–			
High	1.29 (0.95, 1.76)	0.099	1.10 (0.81, 1.49)	0.545	1.11 (0.82, 1.49)	0.506	1.81 (1.33, 2.47)	<0.001	1.00	–			
High All But Umami													
Moderate	0.55 (0.40, 0.75)	<0.001	1.27 (0.94, 1.70)	0.117	0.45 (0.32, 0.62)	<0.001	0.75 (0.55, 1.02)	0.069	0.80 (0.59, 1.10)	0.176	1.00	–	
High	0.95 (0.70, 1.29)	0.731	0.80 (0.60, 1.07)	0.135	0.81 (0.59, 1.11)	0.191	1.33 (0.98, 1.80)	0.067	0.73 (0.54, 0.99)	0.045	1.00	–	
Global p-value[3]													0.350

[1]Values are ORs (95% CI) estimated via multinomial logistic regression, with adjustment for age, sex, physical activity, smoking status, use of antihypertensive and cholesterol lowering medication, type 2 diabetes, and BMI. Alch/Salt/AnimFat, Alcohol, Salty Foods, and Animal Fats dietary pattern.

[2]p-values from Chi Square tests for differences from the reference taste perception profile (calculated by column).

[3]p-value from log likelihood ratio test of joint significance in fully adjusted multinomial logistic regression models. Only 1 p-value is provided, as it is identical for all models regardless of the reference profile.
